# Resting energy metabolism and sweet taste preference during the menstrual cycle in healthy women

**DOI:** 10.1017/S0007114523001927

**Published:** 2024-02-14

**Authors:** Lorena Malo-Vintimilla, Carolina Aguirre, Angie Vergara, Rodrigo Fernández-Verdejo, Jose E. Galgani

**Affiliations:** 1 Departamento de Nutrición, Diabetes y Metabolismo, Facultad de Medicina, Pontificia Universidad Católica de Chile, Santiago, Chile; 2 Carrera de Nutrición y Dietética, Departamento de Ciencias de la Salud, Facultad de Medicina, Pontificia Universidad Católica de Chile, Santiago, Chile; 3 División de Obstetricia y Ginecología, Facultad de Medicina, Pontificia Universidad Católica de Chile, Santiago, Chile; 4 Laboratorio de Fisiología del Ejercicio y Metabolismo (LABFEM), Escuela de Kinesiología, Facultad de Medicina, Universidad Finis Terrae, Santiago, Chile; 5 Pennington Biomedical Research Center, Baton Rouge, LA, USA

**Keywords:** Obesity, Fuel oxidation, Sweet food craving, Appetite, Menopause

## Abstract

Differences in blood concentration of sex hormones in the follicular (FP) and luteal (LP) phases may influence energy metabolism in women. We compared fasting energy metabolism and sweet taste preference on a representative day of the FP and LP in twenty healthy women (25·3 (sd 5·1) years, BMI: 22·2 (sd 2·2) kg/m^2^) with regular self-reported menses and without the use of hormonal contraceptives. From the self-reported duration of the three prior menstrual cycles, the predicted FP and LP visits were scheduled for days 5–12 and 20–25 after menses, respectively. The order of the FP and LP visits was randomly assigned. On each visit, RMR and RQ by indirect calorimetry, sweet taste preference by the Monell two-series forced-choice tracking procedure, serum fibroblast growth factor 21 by a commercial ELISA (FGF21, a liver-derived protein with action in energy balance, fuel oxidation and sugar preference) and dietary food intake by a 24-h dietary recall were determined. Serum progesterone and oestradiol concentrations displayed the expected differences between phases. RMR was lower in the FP *v*. LP (5042 (sd 460) *v*. 5197 (sd 490) kJ/d, respectively; *P* = 0·04; Cohen effect size, *d*_*rm*_ = 0·33), while RQ showed borderline significant higher values (0·84 (sd 0·05) *v*. 0·81 (sd 0·05), respectively; *P* = 0·07; *d*_*rm*_ = 0·62). Also, in the FP *v*. LP, sweet taste preference was lower (12 (sd 8) *v*. 16 (sd 9) %; *P* = 0·04; *d*_*rm*_ = 0·47) concomitant with higher serum FGF21 concentration (294 (sd 164) *v*. 197 (sd 104) pg/ml; *P* < 0·01; *d*_*rm*_ = 0·66). The menstrual cycle is associated with changes in energy expenditure, sweet taste preference and oxidative fuel partitioning.

Oestradiol and progesterone are the primary female sex hormones. Besides their role in reproduction, these hormones appear to influence energy metabolism^(1)^. Thus, pharmacological suppression of progesterone and oestradiol secretion for a short (6 d) and a long (5 months) period decreased RMR in pre-menopausal women^(2,3)^, while oestradiol replacement prevented that effect^(3)^. The extent to which these hormones change from the follicular (FP) to the luteal (LP) phase could elevate RMR, as shown in early studies^(4,5)^. To eliminate the confounding effects of changing hormonal levels, assessing energy expenditure during FP became common practice. However, later studies reported similar RMR along the menstrual cycle^(6,7)^. A systematic review analysed these conflicting findings to determine the difference in RMR between menstrual phases and define the relevance of the phase when measuring RMR^(8)^. The analysis showed lower RMR in the FP *v*. LP, a pattern primarily noted in studies conducted before 2000^(8)^. Notably, most studies qualified as low quality and none as high quality. Lack of objective verification of the menstrual phase, underpowered sample size (<10 subjects) and insufficient control (or poor reporting) of known RMR confounders were the main study flaws^(8)^. The reliability and accuracy of the indirect calorimetry systems to detect changes in RMR may be another factor in play^(9–11)^. Indeed, some metabolic carts have shown insufficient analytical accuracy and reliability^(9,10,12,13)^. This analytical issue led Schadewaldt *et al.* ^(11)^ to propose a method to correct indirect calorimetry data. By simulating VO_2_ and VCO_2_ rates after the subject’s testing through mass-flow regulators and pure gases, the analytical error can be calculated to correct the subject’s indirect calorimetry readouts. We applied that procedure to assess gas exchange in both menstrual cycle phases.

Energy intake also appears to change during the menstrual cycle, increasing from the FP to the LP^(14,15)^. This increase seems higher when energy intake is measured through self- or interview-based food records (∼660−2090 kJ/d)^(16–22)^ compared with objectively measured food intake (∼380 kJ/d)^(23,24)^. Changes in food and macronutrient preferences during the menstrual cycle show a less consistent pattern. Some studies reported lower consumption and preference for sweet foods during the FP than in the LP^(25)^, whereas others did not detect differences^(26,27)^. Inconclusive evidence may result from the subjective nature of these measurements. Using alternative methods, such as sucrose threshold determination (i.e. minimal detectable sucrose concentration), Than *et al*. observed a lower threshold during the FP *v*. LP^(28)^. Whether such a change in the sucrose threshold was associated with a different energy/macronutrient intake or sweet food preferences was not determined.

This study aimed to compare RMR and sweet taste preference between the FP and LP in healthy women. To that end, we assessed RMR, sweet taste preference and energy intake in healthy women on a day representative of their FP (days 5–12) and LP (days 20–25) phases. We also assessed the fasting RQ, an index of the proportion of carbohydrates and fats being oxidised for ATP production. RQ is sensitive to the energy balance and dietary macronutrient composition^(29,30)^, thus representing a valuable tool to estimate changes in these variables along the menstrual cycle. Circulating fibroblast growth factor 21 (FGF21) concentration was also determined. This hepatokine plays a role in energy balance and fuel oxidation^(31)^. FGF21 also suppresses sugar preference in animals and humans^(32,33)^. Importantly, to improve those aspects detected as insufficient in previous studies^(8)^, our design included verification of a biphasic cycle and a more accurate indirect calorimetry system^(10)^.

## Methods

### Participants

Women were recruited by public advertising and invited to a screening visit. They had to be healthy according to face-to-face interviews about their past and current health status. They were excluded if reporting current or history of any of these conditions/diseases: high blood pressure, heart disease, diabetes mellitus, endocrine disease, gastrointestinal disease, dyslipidaemia, gynaecological disease including polycystic ovary syndrome, psychiatric illness and altered food behaviour. Women had to have normal values for routine blood testing, including glucose, urea nitrogen, total bilirubin, Ca, P, total proteins, albumin, cholesterol, thyroid-stimulating hormone, free thyroxine, electrolytes, creatinine, liver enzymes activity and haemogram. Women had regular menstrual cycling (self-reported). Only women with a menstrual cycle of 21 to 35 d (the period between consecutive beginnings of menses) over the last three periods and a maximum variation of 7 d between periods were included. Selected participants were between 18 and 35 years old, had stable body weight (change <3 kg over the past 2 months) and BMI ≥18·5 and <30 kg/m^2^, were non-smokers and did not engage in vigorous physical activity >7 h/week. Participants were not under pharmacological therapy (including hormonal contraceptives such as pills, patches, injections, or intra-uterine devices over the last 3 months) or were not pregnant or lactating. The Ethical Board at Pontificia Universidad Católica de Chile approved the protocol, and participants provided written informed consent before participation.

### Experimental design

**Fig. 1.**
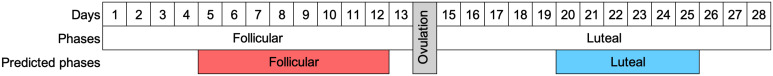
Timeline indicating the periods considered for the predicted follicular and luteal phases. Visits occurred on a representative day of the predicted follicular phase (between days 5 and 12) and the predicted luteal phase (between days 20 and 25).

The menstrual cycle periodicity of all women was registered for 3 months. Then, the first measurement visit was randomly assigned to either the predicted FP or LP. The session for the predicted FP was scheduled on days 5–12 after the menses. The predicted LP session was scheduled for days 20–25 after the menses (Fig. 1). Participants were instructed to avoid vigorous physical activity the day before the measurement visits and maintain their typical dietary pattern. Foods, alcohol, tobacco and caffeine-containing drinks were not allowed for the last 12 h before testing. On the testing day, participants arrived at ∼08.00 hours and were weighed after emptying their bladders. Only participants with a difference in body weight lower than 2·5 kg from the screening visit were allowed to continue the procedure. Afterward, they rested supine for 30 min under thermoneutral and quiet conditions. Blood pressure, body temperature (axillary) and heart rate were determined after 15 min of initiating resting. After the 30-min resting period, gas exchange was determined for 20 min by indirect calorimetry with a canopy system. Once the gas exchange assessment was completed, the accuracy of the calorimetry system was determined, and individual data were corrected as previously reported^(10,11)^. Immediately after the gas exchange assessment (∼09.00 hours), a blood sample was withdrawn to analyse the circulating concentrations of oestradiol, progesterone, insulin, glucose and FGF21. We then determined sweet taste preference by the Monell two-series forced-choice tracking procedure^(34)^. Finally, a dietitian conducted a 24-h dietary recall of the previous day.

### Gas exchange measurement and correction

Gas exchange was determined with a VMax Encore 29n (SensorMedics Co.). The instrument has an IR CO_2_ analyser (±0·02 % accuracy, 0·01 % resolution) and an electrochemical sensor for O_2_ detection (galvanic fuel cell; ±0·02 % accuracy, 0·01 % resolution). The flow rate was adjusted to maintain the fraction of expired CO_2_ between 0·5 % and 1·0 %. The precision of the instrument was 1·1 % for O_2_ consumption (VO_2_) and 1·2 % for CO_2_ production (VCO_2_), calculated from simulations of VO_2_ and VCO_2_ through infusions of N_2_/CO_2_ mixtures.

We performed calorimetric corrections of all gas exchange measurements by determining the accuracy of the VO_2_ consumption and VCO_2_ production analyses. Analytical accuracy was determined by simulating VO_2_ and VCO_2_ exchange through the infusion of pure N_2_ (>99·999 %) and pure CO_2_ (>99·9999 %) into the calorimeter hose using high-precision mass-flow regulators (series 358; 0–2 l/min; Analyt-MTC). The difference between expected and measured VO_2_ and VCO_2_ was used to correct the data of the participants, as previously described^(10)^. RMR (in kJ/d) was calculated as: (3·941 × VO_2_ (l/d) + 1·106 × VCO_2_ (l/d)) × 4.184^(35)^. RQ was calculated as the VCO_2_-to-VO_2_ ratio^(35)^. Gas exchange assessments yielding RQ values equal to or higher than 1·00 were excluded from RMR and RQ analysis.

### Circulating concentrations of hormones and metabolites

Serum progesterone, oestradiol and insulin were measured by the chemiluminescent method, while serum FGF21 was measured by ELISA (R&D systems). Plasma glucose was determined by the glucose oxidase method. The HOMA index of insulin resistance was calculated as described elsewhere^(36)^. Blood progesterone concentration ≥5 ng/ml during the predicted LP was considered indicative of ovulation^(37)^.

### Sweet taste preference

Participants were presented with pairs of solutions with different sucrose concentrations, from 3 % to 36 % w/v. The most preferred sweetness intensity was determined by the Monell two-series forced-choice tracking procedure.^(34)^.

### Dietary energy and macronutrient intake

A dietitian interviewed participants to report their foods/meals and serving size intake for the previous 24 h. Participants were requested to indicate foods and ingredients composing their meals. Food’s energy, macronutrient and total sugars (mono- and di-saccharides) contents were calculated using the USDA Food dataset (https://fdc.nal.usda.gov/). If needed, nutrition facts labels of processed foods were also used. The food quotient was calculated from the relative dietary macronutrient energy composition with a factor of 1·00 for carbohydrates, 0·71 for fat and 0·84 for protein^(38)^.

### Statistical analysis

Data are expressed as mean values and standard deviations. Statistical analyses were performed using SAS software version 9.2 (SAS Institute) or MedCalc® software version 20.118. Raw values were log_10_-transformed or ranked as needed before analyses. Paired Student’s *t* tests were used to assess differences between menstrual phases. Repeated-measures ANOVA (Prox Mixed) was conducted to assess differences between phases according to the presence or absence of ovulation. Thus, a model was developed, including the menstrual phase, presence/absence of ovulation and its interaction. In case of significant interaction, the Tukey test was conducted. Intra-individual variability in RMR was expressed as the sd (in kJ/d). The CV was calculated as described by Fraser and Harris^(39)^ and compared by the Forkman test available in MedCalc® software. The relationship between RMR (in kJ/d) and body weight (in kg) according to menstrual phase was compared by ANCOVA. Statistical significance was set at 5 %, while Cohen’s effect size for repeated measures (*d*_*rm*_) evaluated the clinical significance of differences^(40)^. A *d*_*rm*_ value <0·20, 0·20 to <0·50, 0·50 to <0·80 and ≥0·80 was considered as null, small, moderate and high effect, respectively^(40)^. Based on the intra-individual variability in RMR from a previous study in men^(41)^, we estimated that twenty women would be needed to detect a difference of 293 kJ/d between menstrual phases, considering Cohen’s effect size of 0·60 (90 % power and 5 % type I error).

## Results

Twenty women were included, whose characteristics are shown in Table 1. The interval between the first and second visits was 19 (sd 12) d (range: 10–53). Nine out of twenty women had both visits over the same menstrual cycle, that is, between two menses. Visits for the FP and LP occurred on days 9 (sd 2) (range: 5–12) and 23 (sd 2) (range: 20–25) of the cycle, respectively. In relative terms, the FP and LP occurred at 31 (sd 7) % (range: 19–42) and 78 (sd 8) % (range: 67–91) of their typical menstrual cycle duration, respectively. As expected, FP *v*. LP was characterised by lower serum progesterone (0·3 (sd 0·1) *v*. 6·4 (sd 4·9) ng/ml, respectively; *P* < 0·0001) and oestradiol (85 (sd 61) *v*. 191 (sd 105) pg/ml, respectively; *P* < 0·0001) concentrations. Eight out of twenty (40 %) women had serum progesterone concentration lower than 5 ng/ml in the LP, the cutoff established as indicative of ovulation^(37)^. These women are hereafter referred to as ‘women without ovulation’. Those women still showed lower serum progesterone in FP *v*. LP (0·2 (sd 0·1) *v*. 1·6 (sd 1·2) ng/ml, respectively; *P* < 0·001), but such a difference was about one-fifth of the observed in ovulating women. Of note, the entire group showed the typical lower body temperature in the FP *v*. LP (difference of -0·21 (sd 0·57)°C; *P* = 0·04; *d*_*rm*_ = 0·47), without differences in women with and without evidence of ovulation (-0·21 (sd 0·68) and -0·20 (sd 0·38)°C, respectively; *P* = 0·97). Women with or without evidence of ovulation had no difference in age (*P* = 0·91), BMI (*P* = 0·91), menstrual cycle duration (*P* = 0·75) and the day of their FP and LP visits expressed in either absolute (*P =* 0·71) or relative (*P* = 0·82) terms.

**Table 1.** Characteristics of the participants at the screening visit (*n* 20) (Mean values and standard deviations)

	Mean	sd	Range
Age (years)	25·3	5·1	19·2–34·5
Weight (kg)	58·3	7·1	47·2–73·3
Height (m)	1·62	0·06	1·48–1·73
BMI (kg/m²)	22·2	2·2	19·2–25·7
Menstrual cycle duration (days)	29	3	25–34

For the FP and LP visits, we noted similar body weight (58·4 (sd 7·0) *v*. 58·4 (sd 7·1) kg; *P* = 0·97; *d*_*rm*_ = 0·00), diastolic (*P* = 0·72) and systolic (*P* = 0·11) blood pressure (data not shown), heart rate (*P* = 0·17; data not shown), glycaemia (83·7 (sd 5·1) *v*. 83·3 (sd 4·8) mg/dl; *P* = 0·70; *d*_*rm*_ = 0·08), insulinaemia (4·7 (sd 1·6) *v*. 5·0 (sd 2·0) µmg/ml; *P* = 0·44; *d*_*rm*_ = 0·15) and the HOMA index (0·97 (sd 0·34) *v*. 1·02 (sd 0·39); *P* = 0·48; *d*_*rm*_ = 0·14).

### RMR and RQ

**Fig. 2.**
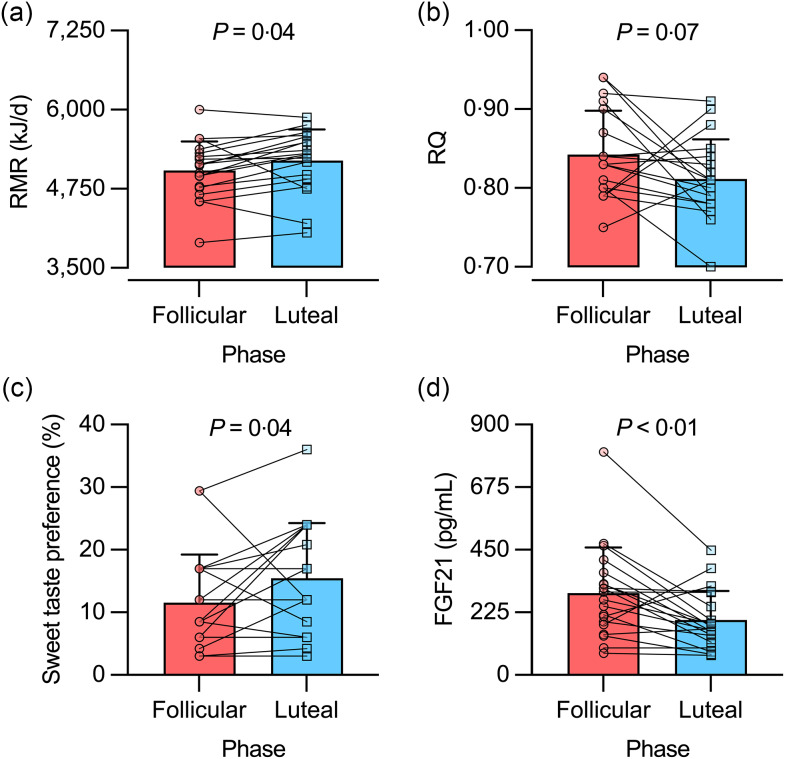
RMR (a), RQ (b), sweet taste preference (c) and serum fibroblast growth factor 21 (FGF21) concentration (d) in the follicular and luteal phases of healthy young women. For statistical analyses, RQ data were ranked, while sweet taste preference and FGF21 data were log10-transformed. Bars represent mean values, including +1 sd. n 19 for RMR and RQ data; n 20 for sweet taste preference and FGF21 data.

We excluded one woman from the gas exchange analysis because her RQ was over 1·00 on the FP visit. Thus, data from nineteen women were considered for analysis. RMR was lower in the FP *v*. LP (5042 (sd 460) *v*. 5197 (sd 490) kJ/d, respectively; *P* = 0·04; *d*_*rm*_ = 0·33; Fig. 2(a)), reaching a RMR difference (ΔRMR, FP – LP) of -155 (sd 310) kJ/d. Such a difference had borderline significance in women with ovulation (ΔRMR: -243 (sd 163) kJ/d; *P* = 0·07; *d*_*rm*_ = 0·79), whereas it was not significant in women without ovulation (ΔRMR: -34 (sd 427) kJ/d; *P* = 0·99; *d*_*rm*_ = 0·05). In the entire group, intra-individual variability in RMR showed an sd of 243 kJ/d (CV = 4·7 %), with similar values in women with and without ovulation (CV = 4·0 and 5·6 %, respectively; *P* = 0·31). Regression analysis of the relationship between RMR (in kJ/d) and body weight (in kg) showed similar slopes (FP *v*. LP, 44 (se 12) *v*. 43 (se 12) kJ/d per kg; *P* = 0·95) and intercepts (FP *v*. LP, 2477 (se 716) *v*. 2703 (se 707) kJ/d; *P* = 0·20) between menstrual phases.

RQ showed borderline significant higher values in the FP *v*. LP (0·84 (sd 0·05) *v*. 0·81 (sd 0·05), respectively; *P* = 0·07; *d*_*rm*_ = 0·62) (Fig. 2(b)). In women without ovulation, RQ was similar in FP *v*. LP (0·85 (sd 0·06) *v*. 0·84 (sd 0·06), respectively; *P* = 0·99; *d*_*rm*_ = 0·20). In turn, women with ovulation showed a pattern similar to the entire group (FP *v*. LP, 0·84 (sd 0·06) *v*. 0·79 (sd 0·04), respectively; *P* = 0·14; *d*_*rm*_ = 1·01).

### Dietary energy, macronutrient and total sugars intake

Energy intake was similar in the FP and LP (5406 (sd 1398) *v*. 5912 (sd 1795) kJ/d, respectively; *P* = 0·34; *d*_*rm*_ = 0·32). Likewise, macronutrient intake was similar between menstrual phases as a proportion of total energy intake. Consequently, the food quotient was similar in the FP and LP (0·88 (sd 0·01) and 0·87 (sd 0·02), respectively; *P* = 0·36; *d*_*rm*_ = 0·31). Similar findings were obtained when considering only women with or without ovulation. Energy intake from total sugars (as % total energy intake) was about 20 %, without differences between phases (*P* = 0·71).

### Sweet taste preference and serum fibroblast growth factor 21 concentration

Sweet taste preference was lower in the FP *v*. LP (12 (sd 8) *v*. 16 (sd 9) %, respectively; *P =* 0·04; *d*_*rm*_ = 0·47; Fig. 2(c)). Such difference was not observed when the analysis was conducted separately in women without ovulation (*P* = 0·24) and with ovulation (*P* = 0·63). In turn, serum FGF21 concentration was higher in the FP *v*. LP (294 (sd 164) *v*. 197 (sd 104) pg/ml, respectively; *P* < 0·01; *d*_*rm*_ = 0·66; Fig. 2(d)). This difference was not observed in women without ovulation (*P* = 0·18; *d*_*rm*_ = 0·57), but it reached borderline significance in women with ovulation (*P* = 0·06; *d*_*rm*_ = 0·79). We repeated the analysis after excluding one participant having the highest serum FGF21 concentration (802 pg/ml in the FP; see Fig. 2(d)). Results remained similar for the entire sample and by sub-groups of women with and without ovulation (data not shown).

## Discussion

Using a calorimetric system of improved accuracy and sample size ∼2-fold larger than often considered^(8)^, we observed that RMR, on average, was lower in the FP *v*. LP in young women with regular menses. Further analysis showed similar regression equations describing the relationship between RMR (kJ/d) and body weight (kg) between phases. We also noted that the between-phase RMR variation was similar to that observed in men over 5 consecutive days^(41)^. Taken together, the difference in RMR between menstrual phases appears small. We also noted a lower sweet taste preference, higher serum FGF21 concentration and a borderline significant higher RQ in the FP compared with the LP. Since body weight remained stable between phases, the menstrual cycle did not affect the balance between energy intake and expenditure. Such a change in fasting RQ suggests a differential preference for carbohydrates over fat as an oxidative source, which might affect food intake. Nevertheless, energy and macronutrient intakes assessed by dietary recalls remained constant during the menstrual cycle.

Whether RMR fluctuates along the menstrual cycle is controversial, a controversy partially explained by the publication date of studies^(8)^. Studies published before 2000 have a ∼2-fold higher likelihood of detecting lower RMR in the FP *v*. LP than those published after 2000. The quality of the metabolic carts may play a role. The low reproducibility of some metabolic carts^(9)^ probably dampens the capacity to detect changes in RMR during the menstrual cycle. In the present study, we improved gas exchange analysis by determining its accuracy after the assessment of each participant and then correcting the measured values^(9,10)^. Thus, our findings on RMR should be independent of analytical issues. The difference in average RMR between phases aligns with a recent, well-performed, novel study that determined the sleeping metabolic rate in a metabolic chamber^(42)^. This study found a lower sleeping metabolic rate in the FP *v*. LP in healthy young women. The difference averaged ∼-335 kJ/d (∼7 %), with a small-to-moderate size effect (*d*_*rm*_ ∼0·50). The lower core body temperature during the FP *v*. LP (-0·27°C) partially accounted for the difference in sleeping metabolic rate. We observed a smaller difference in body temperature (axillary) between phases (-0·21°C), which would have translated into an RMR difference of ∼-251 kJ/d. Thus, the average difference noted in our study represented one-half of the predicted difference. One can speculate that independent and dependent body temperature mechanisms influence RMR during the menstrual cycle. Taken together, menstrual cycle-related RMR variation appears not higher than other sources of variability, including day-to-day variations noted in men^(41,43)^ and circadian variations reported in men and post-menopausal women^(44)^.

Insufficient power to detect changes in RMR may limit the agreement among studies^(8)^. We powered our study to detect a difference of 293 kJ/d between menstrual phases, considering a Cohen’s effect size of 0·60. Such a difference represents the intra-individual CV in RMR often reported in men^(41,43)^. Our study, including a final sample size of nineteen women, allowed detecting a difference in RMR lower than initially planned. Another methodological factor to consider is verifying the menstrual phase in which RMR is assessed. Blood or urinary sex hormone concentrations can be used to confirm the menstrual phase. Not all studies have applied this procedure^(8)^. Our study measured serum sex hormones and used serum progesterone concentration in the predicted LP to verify the occurrence of ovulation^(37)^. Eight out of twenty women had serum progesterone concentrations below the threshold considered suggestive of ovulation (≥5 ng/ml). Still, those eight women showed a statistically different serum progesterone between phases. Eventually, the timing of the testing in non-ovulating and ovulating women could differ, but such a possibility seems unlikely. Indeed, visits occurred at a similar timing of the menstrual cycle in women with and without ovulation. Notably, both sub-groups of women manifested lower body temperature on the FP *v*. LP visits. However, between-phase differences in RMR, RQ and serum FGF21 concentration were accentuated in women with ovulation. Such observation reinforces the notion that menstrual phase influenced those metabolic changes.

An increase in energy intake has been reported in the transition from the FP to the LP^(14,15)^. Those findings often rely on self-records or interview-based food records^(16–22)^, which have poor accuracy, particularly in women^(45)^. We did not detect differences in energy, macronutrient and total sugars intake using an interview-based technique. Food preference changes have also been described during the menstrual cycle, but the evidence is controversial^(25–27)^. We assessed sweet taste preference using a non-memory-dependent method. We also measured fasting RQ, representing an indirect marker of the dietary carbohydrate-to-fat oxidation ratio and energy balance. High-carbohydrate/low-fat diets, positive energy balance or both determine higher fasting RQ^(29,30,46,47)^. We noted a lower sweet taste preference and a trend towards a higher fasting RQ (*P* = 0·07) in the FP compared with the LP. None of these changes was accompanied by changes in the dietary carbohydrate-to-fat ratio or total sugars intake measured in both menstrual phases. Thus, the pattern noted for fasting RQ and sweet taste preference may not have translated into changes in food intake. Alternatively, our interview-based food record assessment could be insufficient to detect such dietary changes.

We measured serum FGF21 concentration due to its role in energy metabolism^(31)^. Humans treated with FGF21 analogues show a decrease in body weight^(33,48,49)^, hepatic fat content^(50)^ and an increase in circulating adiponectin concentration^(33,48–51)^. FGF21 has also been shown to suppress sugar preference in animals^(32,52,53)^, while treatment with an FGF21 analogue reduced energy intake and preference for sweet foods in humans with overweight or obesity^(33)^. Ingestion of sucrose or fructose^(54,55)^ – but not glucose^(55)^ – also increases circulating FGF21 by 2–3 fold. Thus, the notion has emerged that the excess of mono- or di-saccharides increases FGF21 to suppress sugar intake^(56)^. In the present study, the higher serum FGF21 concentration and lower sweet taste preference in the FP *v*. LP appear related. Notably, sweet-disliker *v*. sweet-liker humans showed a ∼1·5-fold higher circulating fasting FGF21 concentration^(55)^. Such a difference in circulating FGF21 concentration is close to the menstrual phase-related difference in the current study. Thus, FGF21 could mediate the change in sweet taste preference between the FP and LP. Whether the change in circulating FGF21 and sweet taste preference during the menstrual cycle has any reproductive relevance is unknown. In this regard, oestradiol enhances hepatic expression and production of FGF21 in female mice^(57)^. As we found lower circulating oestradiol in the FP *v*. LP, we would expect lower circulating FGF21 as well. However, we found the opposite. Then, the changes in circulating FGF21 seem unrelated to those in circulating oestradiol during the menstrual cycle.

We acknowledge limitations in our study, including that we could not confirm the ovulatory status in all women studied. Still, differential patterns observed for RMR, RQ and circulating FGF21 concentration in women with and without ovulation may reinforce our findings. Another limitation, common in studies evaluating energy and macronutrient intake, is the assessment method based on food intake reports. Finally, our evaluation conducted under fasting conditions would have benefited from a supervised control of the 24–48 h period before assessment.

In conclusion, RMR fluctuates along the menstrual cycle in healthy young women. However, such variation is similar to day-to-day variations described in men or post-menopausal women. Sweet taste preference and fasting fuel oxidation appear sensitive to the transition from the FP to LP. Standardisation of the menstrual phase is preferable for studies involving RMR assessment, especially when fuel oxidation and food behaviour are relevant measurements.
